# Photosynthetic Electron Transport in Winter Wheat: Responses to Low-Temperature and Weak-Light Condition

**DOI:** 10.3390/cells14161275

**Published:** 2025-08-18

**Authors:** Cheng Yang, Minghan Liu, Simeng Du, Deqi Zhang, Xiangdong Li, Liting Wu, Yanhua Shi, Baoting Fang, Ge Yan, Fang Wei

**Affiliations:** 1 Wheat Research Institute, Henan Academy of Agricultural Sciences, Postgraduate T&R Base of Zhengzhou University, Zhengzhou 450002, China18595331582@163.com (M.L.); zhangdeqi@hnagri.org.cn (D.Z.); wjw012102@163.com (L.W.);; 2 School of Agricultural Sciences, Zhengzhou University, Zhengzhou 450052, China

**Keywords:** wheat, photosynthesis, low temperature, weak light, photosystem II, photosystem I

## Abstract

Spring low temperatures are a serious natural threat to wheat production in the Huang-Huai wheat region, and they are often accompanied by weak light environments during the day. To elucidate the response patterns and adaptation mechanisms of winter wheat leaves to low-temperature and weak-light environments, we simultaneously measured prompt chlorophyll a fluorescence, delayed chlorophyll a fluorescence, and modulated 820 nm light reflection; moreover, we analyzed the effects of low temperature and weak light treatment for different duration (2 h and 4 h) on the donor-side activity of photosystem II (PSII), the degree of PSII unit dissociation, the efficiency of light energy absorption and capture by PSII, electron transfer to Q_A_^−^ and PSI terminal, PSI activity and cyclic electron transport activity in isolated wheat leaves under controlled conditions. The results, which were corroborated using the three methods, revealed that in low-temperature and weak-light environments, the degree of PSII unit dissociation, and the efficiency of light energy absorption, capture, and electron transfer to Q_A_^−^ decreased, while the donor-side activity remained unaffected. In contrast, the efficiency of electron transfer to the PSI terminal and the overall performance of photosynthetic electron transport increased. Comprehensive analysis suggests that the increase in the electron receptor pool at the PSI terminal under low-temperature stress is a crucial factor contributing to the enhanced electron transfer efficiency to the PSI terminal and the improved overall performance of the photosynthetic electron transport chain, which is also a crucial factor in the high cold tolerance of winter wheat.

## 1. Introduction

Wheat is one of the three major staple crops in China, serving as the primary food source for most people in northern China. Henan Province, a crucial production area for winter wheat in China, contributes approximately 28% of the country′s total wheat yield and accounts for about 20% of the total wheat cultivation area. The stability of wheat production in Henan Province plays a vital role in safeguarding national food security. Located at the transitional zone between the warm temperate and subtropical regions, Henan Province experiences a complex and diverse climate, making it particularly susceptible to meteorological disasters [1]. Since the 21st century, global temperature rises have further exacerbated the frequency and severity of natural disasters [2,3]. As one of the most detrimental natural disasters affecting wheat production, spring low temperatures have long been a focal point for agricultural and meteorological experts, as well as researchers in these fields.

As the temperature increases in spring, wheat rapidly enters the ear differentiation stage, during which its sensitivity to low temperatures gradually increases. Alt et al. found that young wheat ears exhibit the lowest sensitivity to low temperatures during the double-ridge stage, reaching peak sensitivity during the anther separation stage [4]. Low temperatures during the anther stage not only led to floret abortion and a reduction in the number of grains per spike but also caused significant decreases in the length, width, and thickness of caryopses, while adversely affecting the development of starch granules in endosperm cells [5]. Although spring low temperatures generally result in reduced wheat yields, variations in temperature variations could lead to differences in yield components. Research by Xiang et al. indicated that low temperatures above freezing (2 °C) primarily reduce the number of grains per spike, whereas temperatures below 0 °C could lead to an increase in grain weight due to a significant reduction in the number of grains per spike [6]. Spring low temperatures not only directly affected the ear development process but also indirectly influenced later stages of ear development and grain filling by impacting the photosynthetic apparatus. Liu et al. found that although the activity of antioxidant enzymes in the leaves could be induced and significantly increased under low-temperature stress, the chloroplast structure of functional wheat leaves is still damaged, leading to membrane structure impairment, chlorophyll degradation, grana lamella disintegration, and a significant reduction in photosynthetic rate [7]. Research by Zhang et al. demonstrated that while the photosynthetic function of leaves could gradually recover within 7–15 days after short-term treatment exposure to 2 °C or −2 °C, prolonged treatment at −2 °C for 72 h could cause irreversible damage to the photosynthetic apparatus [8].

Photosystem I (PSI) and photosystem II (PSII) are two critical components of the photosynthetic electron transport chain. Their sensitivity to low temperatures changes with light intensity. PSII is more susceptible to inhibition or damage under strong light [9]. Damage to PSII reduces electron transfer from PSII to PSI, thereby alleviating over-excitation of PSI and the formation of reactive oxygen species (ROS) at the PSI terminal and preventing photoinhibition or damage to PSI [10,11]. After low-temperature stress is removed, PSII can be rapidly repaired through D1 protein turnover, which rapidly recovers photosynthetic electron transport capacity. However, under low-temperature and weak-light conditions, while carbon assimilation is inhibited, PSII is less likely to be damaged, leading to an increase in the flow of electrons to PSI. This accelerates the production of ROS at the PSI terminal and the over-excitation of PSI, exacerbating the damage to PSI [12]. During spring cold waves, weak-light conditions due to overcast or rainy weather frequently occur during the day. Previous research on photosynthesis under low-temperature, weak-light conditions has primarily focused on facility-grown vegetables, such as cold-sensitive plants like cucumbers and tomatoes [11,13]. As an overwintering plant, wheat has greater low-temperature resistance in its photosynthetic apparatus than most other plants. However, to date, it remains unclear how the photosynthetic electron transport process in wheat, particularly at different stages of electron transport, responds to low-temperature and weak-light environments, and whether PSI undergoes specific photoinhibition under such conditions, as observed in cold-sensitive plants.

Chlorophyll fluorescence technology and its related equipment are generally employed in agricultural, environmental, and life sciences research. Despite some advantages, such as portability, rapid measurement, and non-destructive testing, this technology also has certain limitations [14]. As effective complements to chlorophyll fluorescence technology, MR_820nm_ light reflection and delayed fluorescence techniques have increasingly been applied in studies related to plant-stress photosynthesis in recent years [14,15,16]. Therefore, this study aims to use synchronized measurements of rapid chlorophyll fluorescence, 820 nm light reflection, and delayed fluorescence to investigate the effects of different durations of low-temperature and weak-light exposure on the photosynthetic electron transport in the most recent fully expanded leaves of wheat during the heading stage. The findings will provide a theoretical basis for breeding cold-resistant wheat varieties and innovating spring management techniques.

## 2. Materials and Methods

### 2.1. Materials and Experimental Design

**Figure 1:**
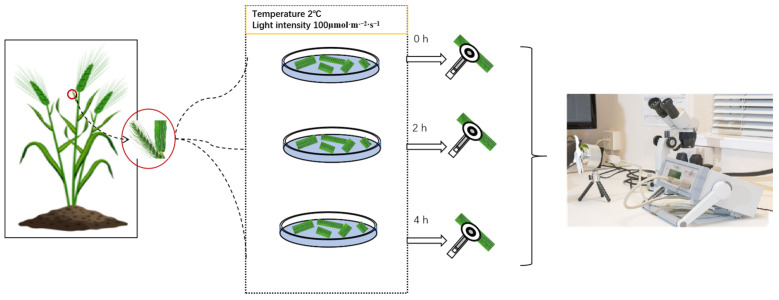
The flow chart of the experiment. The leaf fragments of the flag leaves were floated on the surface of 10 mL of purified water in a 9 cm diameter Petri dish after being cut from the plant. Following treatment for various durations (0 h, 2 h, 4 h), leaf activity was measured using a multifunctional plant efficiency analyzer (M-PEA) with a program that synchronized the measurements of PF, MR, and DF.

The experiment used the nationally approved wheat variety Zhengmai 20 as the experimental material. The wheat was cultivated in the experimental base of the Henan Academy of Agricultural Sciences in Yuanyang county, Henan Province. The soil type in the experimental field is high in organic matter and is a slightly alkaline sandy clay. The experiment was conducted in a large planting area of 90 m^2^. The experimental field had uniform fertility and was prepared according to standard agricultural practices. Seeding was carried out on 12 October 2020, at a rate of 150 kg/ha and with a row spacing of 0.2 m, utilizing a plot planter. Throughout the entire growth phase, the field was managed with consistent and rational practices, encompassing timely fertilization and irrigation. During the booting stage (29 March 2021), the latest fully expanded, uniform, and intact leaves, were collected before 8:00 AM. The leaves were wrapped in wet gauze and transported to the laboratory. Following the method described by Yang [15], the bases and tips of the leaves were trimmed, retaining the middle section of 2–3 cm. The leaf segments were placed adaxial side up on the surface of 10 mL of purified water in a 9 cm diameter Petri dish. The dishes were then placed in a low-temperature artificial climate chamber (QLRX-450D-3000, Shanghai Qizhan Experimental Equipment Co., Ltd., Shanghai, China.) set at 2 °C, and whit a light intensity of 100 μmol·m^−2^·s^−1^. Measurements of rapid chlorophyll fluorescence induction kinetics, 820 nm light reflection, and delayed fluorescence were conducted at 0 h, 2 h, and 4 h of treatment. Each treatment included six replicate leaf samples. The flow chart of the experiment is shown in Figure 1.

### 2.2. Measurement of Rapid Chlorophyll Fluorescence Induction Kinetics, 820 nm Light Reflection, and Delayed Fluorescence

The measurements were performed using a multifunctional plant efficiency analyzer (M-PEA, Hansatech Instruments Ltd., King’s Lynn, Norfolk, UK) following the method described by Yang et al. [15]. Leaves were dark-adapted for 30 min before measurements. A 2-s red light pulse at 5000 μmol·m^−2^·s^−1^ was used to simultaneously record the rapid chlorophyll fluorescence induction kinetics (OJIP), delayed fluorescence (DF), and 820 nm light reflection (MR). The wavelength of the red light was (627 ± 10) nm. During the synchronized measurements, light-to-dark transitions began at 300 μs after exposure. The rapid fluorescence and 820 nm light reflection signals were recorded under light conditions, while the delayed fluorescence signals were recorded in the dark. The entire measurement process lasted for 2 s.

The chlorophyll fluorescence induction kinetics were normalized using the method described by Goussi et al. [17], as follows (Table 1). Normalization of the O-P phase: V_t_ = (F_t_ − F_O_)/(F_M_ − F_O_). Normalization of the O-J phase: V_K_ = (F_t_ − F_O_)/(F_J_ − F_O_). Normalization of the O-K phase: V_L_ = (F_t_ − F_O_)/(F_K_ − F_O_). Secondary normalization: ΔV = V_treatment_ − V_control_.

### 2.3. Statistical Analysis

Data processing and analysis were performed using Microsoft Office 365 (Excel) and Data Processing System (Hangzhou RuiFeng Information Technology Co., Ltd., Hangzhou, China) V18.10 software [18,19,20], The least significant difference (LSD) multiple comparison method (*p* < 0.05) was used for statistical comparisons. Graphs were generated using Sigma Plot 12.0 software.

## 3. Results

### 3.1. Effects of Low Temperature on Chlorophyll Fluorescence Induction Kinetics Curves

**Figure 2:**
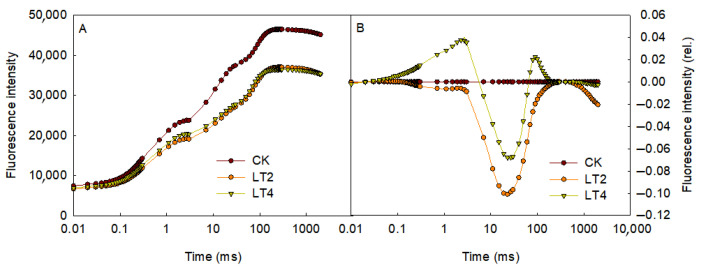
The effects of low temperature on rapid-chlorophyll-fluorescence kinetics curves of wheat leaves. (**A**): Original rapid chlorophyll-fluorescence curve; (**B**): Curves after secondary normalization of the O-P phase. All curves represent the average measurement from six leaves.

The chlorophyll fluorescence induction kinetics curves of detached wheat leaves were measured after exposure to 0 (CK), 2 (LT2), and 4 (LT4) hours of low temperature and weak light. As shown in Figure 2A, after 2 h of low-temperature treatment, the maximum fluorescence intensity at point p (Fm) showed a significant decrease compared to the control. However, there was no significant difference between 4 h and 2 h of low-temperature treatments. Following the method described by Yang et al. [15], the O-P phase was subjected to secondary normalization (subtracting the normalized O-P values of the control), after 4 h of low-temperature treatment, point J showed a significant increase, while point I decreased after 2 h of low-temperature treatment.

**Figure 3:**
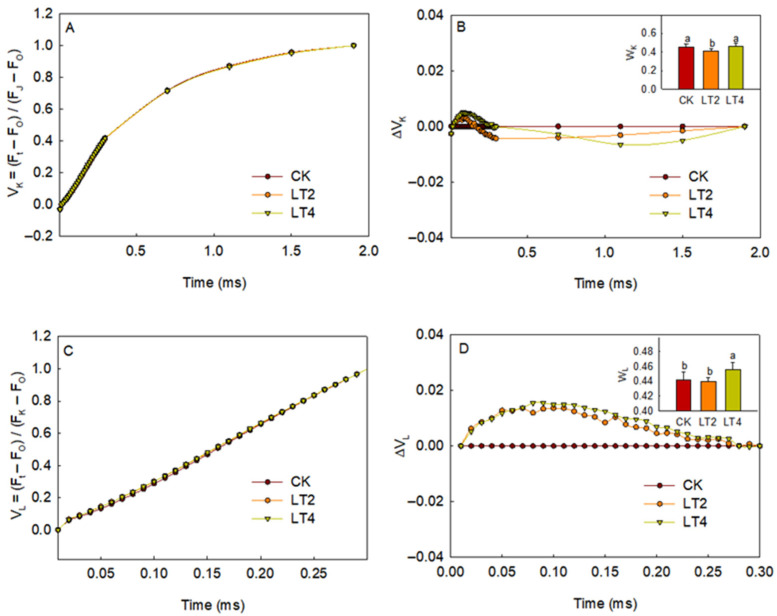
The impact of low temperature and weak light on the O-J and O-K phases of fluorescence in wheat leaves. (**A**,**C**) The normalized curves of the O-J (**A**) and O-K (**C**) phase in leaves treated for different amounts of time. (**B**,**D**) The second normalized curves of the O-J (**B**) and O-K (**D**) phases in leaves treated for different amounts of time, expressed as ΔVt = Vt ^LT2 or LT4^ − Vt ^CK^. The W_K_ and W_L_ parameters for leaves under different treatments are shown in the insets. Each curve (or datapoint) is the average from 6 leaves. The values of the inner figures are presented as means ± SD (*n* = 6). The least significant difference (LSD) multiple comparison method (*p* < 0.05) was used for statistical comparisons. Different letters represent statistically significant differences (*p* < 0.05) among the different treatments.

To obtain more information about PSII, the O-J phase of the rapid-chlorophyll-fluorescence curve was normalized, allowing clear observation of fluorescence changes at 300 μs. The fluorescence at 300 µs is commonly used to reflect the activity of the donor side of PSII [21,22]. As shown in Figure 3A, no differences were observed between the low-temperature treatment and the control after normalizing only the O-J phase (Figure 3A). However, after secondary normalization, the fluorescence at point K did not increase but instead showed a slight decrease after 2 h of treatment, indicating that the donor side activity of PSII was not significantly inhibited by low temperature (Figure 3B). Next, the O-K phase of the fluorescence curve was normalized using the same method (Figure 3C,D), revealing fluorescence changes at 150 µs, also known as point L. After secondary normalization, it was observed that the low-temperature exposure altered the shape of the fluorescence curve, with point L showing a significant increase after 2 h (Figure 3C,D). The fluorescence at point L is commonly used to indicate the energy coupling between PSII units [21,23,24]. The results indicated that the degree of dissociation among PSII units increased significantly after 2 h of low-temperature treatment.

**Figure 4:**
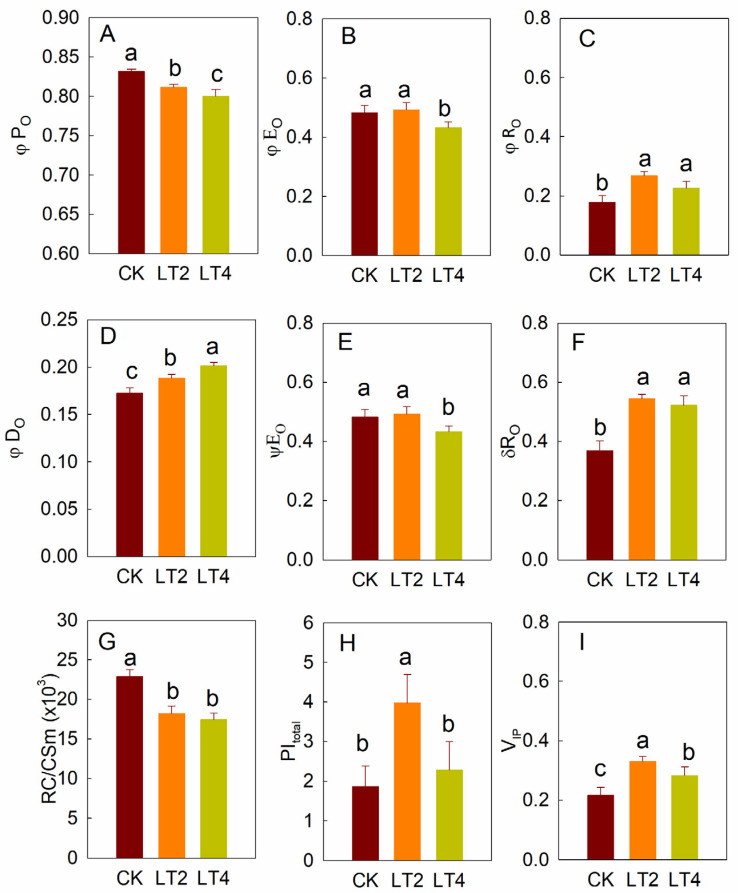
The effects of low temperature on chlorophyll fluorescence parameters. (**A**–**C**) φP_O_ (**A**), φE_O_ (**B**), and φR_O_ (**C**) in wheat leaves treated for different amounts of time, (**D**–**F**) φD_O_ (**D**), ψE_O_ (**E**) and σR_O_ (**F**) in wheat leaves treated for different amounts of time, (**G**–**I**) RC/CS_m_ (**G**), PI_total_ (**H**) and V_IP_ (**I**) in wheat leaves treated for different amounts of time. Different letters above the bars indicate significant differences among the treatments at the *p* < 0.05 level. CK, LT2, and LT4 mean leaves treated in low temperature and weak light for 0, 2, and 4 h, respectively. The values were presented as means ± SD (*n* = 6). The least significant difference (LSD) multiple comparison method (*p* < 0.05) was used for statistical comparison. Different letters represent statistically significant differences (*p* < 0.05) among different treatments.

To better characterize information related to photosynthetic electron transport, a series of fluorescence parameters based on key characteristic points of the rapid-chlorophyll-fluorescence curve was calculated. After 2 h of low-temperature treatment, although φP_O_ remained above 0.8, it was significantly decreased compared to the control (Figure 4A); moreover, the decrease was more pronounced after 4 h of treatment, suggesting a gradual decline in the efficiency of light energy capture by PSII under low-temperature and weak-light conditions. φE_O_ showed a significant decrease after 4 h of low-temperature treatment compared to the control (Figure 4A,B). φE_O_ was significantly less sensitive than φP_O_, only showing a significant decrease only after 4 h of low-temperature treatment (Figure 4A,B). In contrast, φR_O_ exhibited the opposite trend, showing a significant increase after 2 h of low-temperature treatment. φD_O_ gradually increased as the low-temperature treatment was prolonged (Figure 4D). ψE_O_ and σR_O_ showed trends similar to those for φE_O_ and φR_O_, respectively (Figure 4E,F). RC/CS_m_, which reflects the number of active PSII reaction centers per unit area, showed a significant decrease after 2 h of low-temperature treatment (Figure 4G). Although it remained significantly lower than the control after 4 h, there was no significant difference compared to the 2 h treatment (Figure 4G). PI_total_ significantly increased after 2 h of low-temperature treatment but returned to baseline levels after 4 h (Figure 4H). V_IP_, the relative change in fluorescence in the I-P phase of the fluorescence curve, showed a significant increase after 2 h of low-temperature treatment and a subsequent decrease after 4 h, although it remained significantly higher than that in the control (Figure 4I).

### 3.2. Effects of Low Temperature on MR_820nm_ Light Reflection

**Figure 5:**
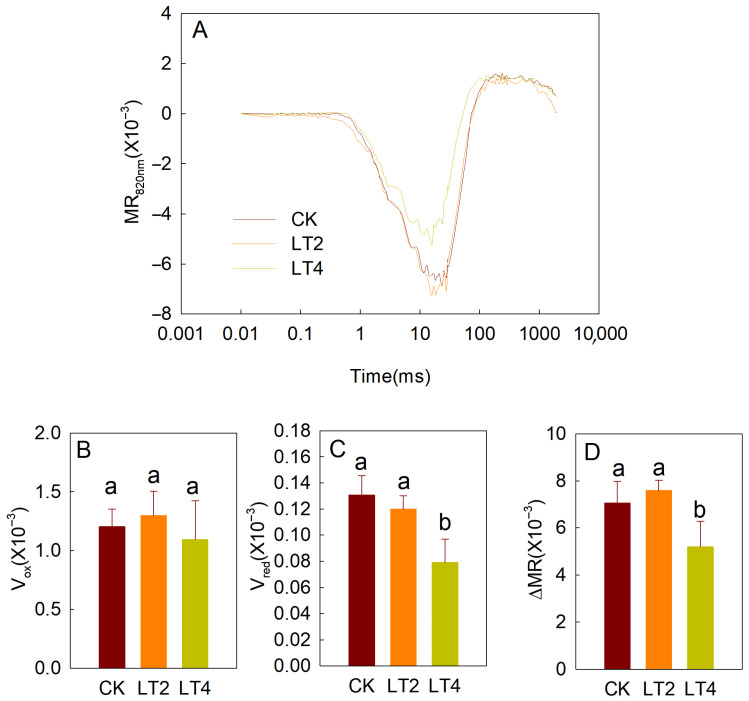
Effects of low temperature on parameters of MR_820nm_ induction curves and parameters in wheat leaves. (**A**) MR_820nm_ induction curves of leaves treated for different amounts of time. Each curve (or datapoint) represents the average from 6 leaves. (**B**–**D**) V_OX_ (**B**), V_RED_ (**C**), and ∆MR (**D**) in wheat leaves treated for different amounts of time. The values are presented as means ± SD (*n* = 6). The least significant difference (LSD) multiple comparison method (*p* < 0.05) was used for statistical comparisons. Different letters represent statistically significant differences among different treatments.

The reaction centers of PSI, P700, and plastocyanin (PC) can transfer electrons downstream upon illumination, while being oxidized to P700^+^ and PC^+^. P700^+^ and PC^+^ specifically absorb 820 nm light, allowing the monitoring of changes in the reflectance of 820 nm light by leaves to reflect the accumulation state of P700^+^ and PC^+^. In the initial phase of saturating red light illumination on fully dark-adapted leaves, electrons from PSII have not yet been transferred to PSI; as a result, P700^+^ and PC^+^ rapidly accumulate, causing a sharp decrease in 820 nm light reflection. The maximum slope of this decrease (V_ox_) typically represents the maximum oxidation rate of PSI and PC [21,25]. As illumination continues, P700 and PC oxidation reaches its peak and the MR curve no longer decreases, attaining an equilibrium point. The maximum amplitude of this decrease typically represents the maximum activity of PSI (ΔMR). Subsequently, as more electrons are transferred from the donor side, P700^+^ and PC^+^ are rapidly reduced, causing the MR to rise quickly. The maximum slope of this rise is usually associated with activity of PSII [15]. From the normalized curves (Figure 5A), it can be observed that the MR curve after 2 h of low-temperature treatment showed no significant difference compared to the control. However, after 4 h of low-temperature treatment, the MR curve showed significant differences compared to both the control and the 2 h treatment. By comparing specific parameters (Figure 5B–D), it was found that 2 h of low-temperature treatment had no significant effect on V_OX_, ΔMR, or V_red_. In contrast, 4 h of low-temperature treatment resulted in significantly lower MR and V_red_ compared to the control and the 2 h treatment.

**Figure 6:**
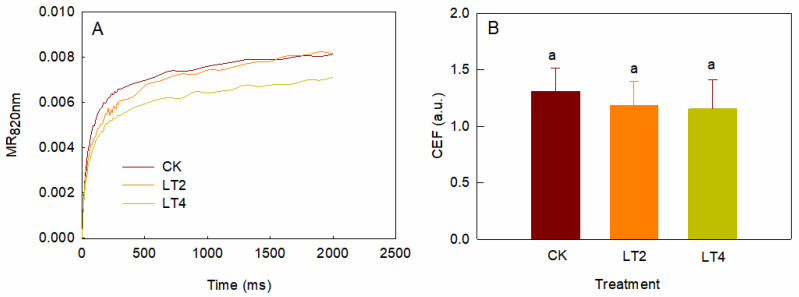
Effects of low temperature on the cyclic electron transport of leaves treated for different times. (**A**) The increasing curves of MR_820nm_ after far-red light was removed. Each curve represents the average from 6 leaves. (**B**) The initial increasing rate of the MR_820nm_ curves obtained after far-red light was removed. The values are presented as means ± SD (*n* = 6). Different letters represent statistically significant differences (*p* < 0.05) among different treatments.

Cyclic electron transport can transfer electrons from PSI terminals back to PQ, forming a cyclic electron flow around PSI. In dark-adapted leaves, the re-reduction process of PSI can be monitored after far-red light illumination is removed (Figure 6A), and the initial rate of PSI re-reduction is regarded as an indicator of the strength of cyclic electron transport [11,26]. As shown in Figure 6, the initial rate of PSI re-reduction after 2 or 4 h treatment did not differ significantly from that of the control after 4 h of low-temperature treatment (Figure 6B), suggesting that cyclic electron transport was not changed after 4 h of low-temperature and weak-light exposure.

### 3.3. Effects of Low Temperatures on Delayed Fluorescence in Wheat Leaves

During the measurement of instantaneous fluorescence curves, the light is repeatedly turned off, and the fluorescence intensity at the same time points after each light-off event is connected to form the induction kinetics curve of delayed fluorescence [26,27,28]. Figure 6A shows the 20 µs delayed fluorescence induction kinetics curves obtained for the control and after 2 and 4 h of low-temperature treatment. Three distinct characteristic points, I_1_, I_2_, and I_3_, can be observed at 7 ms, 100 ms, and 1000 ms, respectively. In contrast to the stability of I_2_, I_1_ decreases, causing the I_2_/I_1_ ratio to be significantly higher than the control in response to low-temperature treatment (Figure 6A). After each light-off event, the delayed fluorescence intensity gradually decreases; the curve formed by connecting these fluorescence signals is referred to as the delayed fluorescence decay curve [26,27,28]. As shown in Figure 6, the decay rate of fluorescence at point I_1_ was significantly lower in the low-temperature-treated samples compared to the control. Using the method described by Gao et al., the delayed fluorescence decay curve at point I_1_ was deconvoluted to obtain five parameters: L_1_, L_2_, L_3_, τ_1_, and τ_2_. L_1_, L_2_, and L_3_ represent the intensities of different delayed fluorescence components, while τ_1_ and τ_2_ represent the lifetimes of the L_1_ and L_2_ components, respectively [27]. As shown in Table 2, L_1_ and L_2_ correspond to the 20 µs and 300 µs delayed fluorescence components, respectively. The 20 µs delayed fluorescence component dominates at point I_1_, significantly exceeding the 300 µs component. After 2 h of low-temperature treatment, both L_1_ and L_3_ were significantly lower than the control, and there was no significant difference between the 4 h and 2 h treatments.

## 4. Discussion

Light energy is the source of energy in plant photosynthesis. Under favorable environmental conditions, plants can synthesize the carbohydrates required for growth and development through photosynthesis. However, under stress conditions, light can accelerate the production of reactive oxygen species (ROS) in the photosynthetic electron transport chain, exacerbating the stress [11,29]. To investigate how photosynthetic electron transport in winter wheat responds to low temperature and weak light, we used synchronized measurements of rapid chlorophyll fluorescence, MR_820nm_, and delayed fluorescence to analyze changes in the photosynthetic electron transport process of the most recent fully expanded leaves of winter wheat during the booting stage.

The chlorophyll-fluorescence induction-kinetics curves contains rich information about photosynthesis, particularly regarding the photosynthetic electron transport process, and these curves have been widely used in studies related to various biotic and abiotic stress [26,30,31,32]. In this study, a significant decrease in the fluorescence intensity at point P was observed after 2 h of low-temperature treatment (Figure 2). The decline in point Pt fluorescence may be attributed to two factors: (1) the inactivation of PSII reaction centers, where the absorbed energy is dissipated as heat, leading to a reduction in maximum fluorescence [33,34], and (2) chlorophyll degradation resulting in a decrease in chlorophyll fluorescence intensity [35,36]. Since chlorophyll degradation would also be reduce the fluorescence intensity at the point O, and no significant change at point O was observed in Figure 2A, we speculate that the decrease in point P fluorescence is primarily due to an increase in inactivated reaction centers. This inference is supported by the observed increase in thermal dissipation efficiency (φD_O_), the decrease in the maximum photochemical efficiency of PSII (φP_O_), and the reduction in the number of active reaction centers per unit area (RC/CS_m_) under low-temperature stress (Figure 4). The activity of the receptor side is a crucial factor influencing PSII activity. Inhibition of electron transport on the receptor side can lead to over-excitation of PSII reaction centers and accelerated production of reactive oxygen species, further aggravating photoinhibition [37,38,39]. In this experiment, after 4 h of low-temperature treatment, the rise in point J and decrease in the efficiency of electron transfer from PSII to Q_A_ (Figure 2; Figure 4E) indicate that electron transfer from Q_A_ to Q_B_ on the receptor side of PSII was inhibited. The activity of the donor side is also a crucial factor influencing PSII photoinhibition. Damage to the donor side typically results in a specific increase in fluorescence at 300 µs (Figure 3A, B), making the point K fluorescence a common indicator of donor side damage. In this study, no significant increase in the K point was observed after low-temperature treatment, indicating that the donor side of PSII remained unharmed. Previous research has shown that PSII activity is more sensitive to high-temperature stress, while low temperatures does not damage the donor side of PSII, further supporting this conclusion. The intensity of the microsecond-level delayed fluorescence component S3Z^+^P680Q_A_^−^Q_B_ is influenced by the activity of the PSII reaction center, the donor side activity, and the receptor side activity [14]. The suppression of the S3Z^+^P680Q_A_^−^Q_B_ component after low-temperature treatment (I_1_ and L_1_) was primarily due to the inhibition of active reaction centers and electron transfer from Q_A_ to Q_B_, as the oxygen-evolving complex was not significantly damaged. This further confirms the results obtained from rapid-chlorophyll-fluorescence measurements.

The fluorescence at point L reflects the energy exchange between PSII units. An increase in point L fluorescence indicates the dissociation of PSII units [40,41]. It is also suggested that an increase in point L fluorescence might be a marker for thylakoid lamellar structure destruction in chloroplasts [42]. Liu Luzhou et al. analyzed the ultrastructure of winter leaves after low-temperature treatment and found disintegration of the grana lamellae [7]. Therefore, the significant increase in L point fluorescence after low-temperature treatment in our study indicates dissociation among PSII units, likely due to structural damage to the thylakoid (Figure 3).

**Figure 7:**
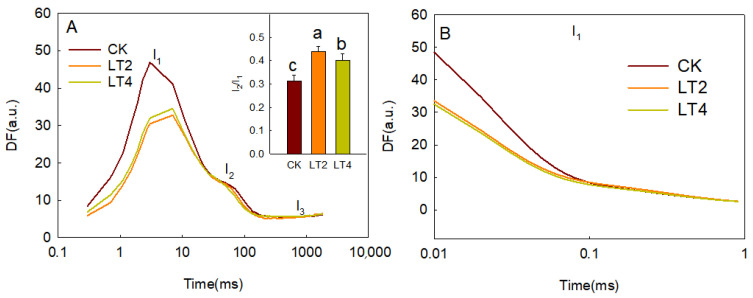
Effects of low temperature on delayed fluorescence in wheat leaves. (**A**) DF induction kinetics at 20 µs. The I_2_/I_1_ parameters for leaves in response to different treatments are shown the inset. (**B**) DF decay curves at I_1_ in leaves treated for different amounts of time. Each curve (or datapoint) represents the average from 6 leaves. The values of the inner figures were presented as means ± SD (*n* = 6). The least significant difference (LSD) multiple comparison method (*p* < 0.05) was used for statistical comparisons. Different letters represent statistically significant differences (*p* < 0.05) among different treatments.

Different stages of photosynthetic electron transport have different sensitivities to stress. For instance, high-temperature stress often causes more severe damage to the donor side of PSII [43,44], while in cold-sensitive plants, low-temperature and weak-light stress primarily damages PSI [45,46]. In this study, the comprehensive performance parameter of photosynthetic electron transport, PI_total_, exhibited a significant increase after 2 h of low-temperature treatment. Since PI_total_ reflects the overall performance of light energy absorption by PSI to electron transfer to the PSI terminal, and both the light energy capture efficiency of PSII and the efficiency of electron transfer to downstream Q_A_ decreased under low-temperature conditions, the rise in PI_total_ is primarily attributed to a significant increase in the efficiency of electron transfer to the PSI terminals (φR_O_, σR, and V_IP_) (Figure 4). Although low-temperature stress inhibited the light-harvesting efficiency of PSII and the electron transfer to Q_A_, the electron transfer efficiency to PSI terminal significantly increased, displaying notable differences after only 2 h of low-temperature treatment. This conclusion is further supported by the increase in the delayed fluorescence ratio I_2_/I_1_ (Figure 7). Based on previous research, there are three possible explanations for this phenomenon: (1) an increase in PSI activity [47]; (2) an increase in electron acceptors at the PSI terminal [48]; or (3) an increase in cyclic electron transport [30]. However, in our experiment, cyclic electron transport was not significantly altered after treatment (Figure 6), and PSI activity (ΔMR) significantly decreased after 4 h of low-temperature treatment (Figure 5). Therefore, we hypothesize that an increased efficiency of electron transfer from the PSII to PSI terminals under low-temperature stress might be due to an increase in the pool of terminal electron acceptors. Previous studies on cold-sensitive plants, such as tomatoes, have shown significant decreases in I-P phase fluorescence after in response to low-temperature stress, with similar trends observed during autumn-winter transitions [30]. Thus, we further speculate that the increase in the pool of electron acceptors at the PSI terminal might contribute to strong cold resistance of wheat. However, further research is needed to specifically identify which electron acceptors at the PSI terminal are increased.

Although wheat is an overwintering plant with strong cold resistance, significant variations exist in cold tolerance among different wheat varieties. Fang et al. evaluated the cold resistance in 11 wheat varieties from the Huang-Huai wheat region and found significant variability among them [4]. By measuring the soluble sugar content, antioxidant enzyme activity, and yield indicators—and using the membership function method—the varieties were classified into four cold-resistant, and moderately cold-resistant groups. Guan et al. compared the effects of low temperatures at the jointing stage on the photosynthetic rates of different cold-resistant varieties and found that the photosynthetic performance of the semi-winter variety Yannong 19 was significantly higher than that of the weak-spring varieties Yangmai 18 and Zhengmai 9023 after low-temperature stress [49]. Zhang et al. observed that after 72 h of treatment at −2 °C, the cold-sensitive variety Xinmai 26 showed not only lower photosynthetic capacity than Yannong 19 but also failed to recover to normal levels [8]. Therefore, further research is needed to determine whether the response characteristics of photosynthetic electron transport to low temperature are consistent across different wheat varieties and whether the size of the electron receptor pool at the PSI terminal is a crucial factor that can influence varietal differences in cold resistance and the final yield. Since this is the first time that this phenomenon has been found in wheat, whether this discovery is universal or not, it will provide an important reference for the studying spring frost damage in wheat.

## 5. Conclusions

In this study, we employed synchronized measurements of rapid chlorophyll fluorescence, MR_820nm_ light reflection, and delayed fluorescence to investigate the response of the spring leaves of winter wheat to low-temperature and weak-light conditions. Our results, corroborated using the three methods, indicated that exposure to low temperatures led to a decrease in the efficiency of light energy absorption, capture, and electron transfer to Q_A_^−^ by photosystem II (PSII), as well as the dissociation of PSII units. However, the activity of the oxygen-evolving complex was not significantly inhibited. Notably, the efficiency of electron transfer to the PSI terminal and the overall performance of the photosynthetic electron transport processes significantly improved. Based on cyclic electron transport and PSI activity measurements, we hypothesize that the pool of electron acceptors and PSI terminal may be a critical factor in the enhanced efficiency of electron transfer from PSII to PSI terminal, as well as the overall improvement in photosynthetic electron transport performance.

## Figures and Tables

**Table 1:** Parameters and formulas of rapid chlorophyll-fluorescence induction–kinetic curves.

Parameter	Method of Calculation
F_M_	Maximum fluorescence intensity obtained under light after dark adaptation
F_O_	Fluorescence intensity at 20 μs of OJIP curve
F_v_	F_v_ = F_M_ − F_O_
F_t_	Fluorescence intensity at t time
F_K_	Fluorescence intensity at the K point (0.3 ms)
F_J_	Fluorescence intensity at the J point (3 ms)
F_I_	Fluorescence intensity at the I point (30 ms)
V_J_	V_J_ = (F_J_ − F_O_)/(F_M_ − F_O_)
V_I_	V_I_ = (F_I_ − F_O_)/(F_M_ − F_O_)
M_O_	M_O_ = 4(F_300μs_ − F_O_)/(F_M_ − F_O_)
Ψ_O_	ψ_O_ = 1 − V_J_
φP_O_	φP_O_ = F_v_/F_M_ = (F_M_ − F_O_)/F_M_
φE_O_	φEo = ETo/ABS = [1 − (F_O_/F_M_)] × ψ
φD_O_	φD_O_ = 1 − φP_O_
φR_O_	φR_O_ = φP_O_ × (1 − V_I_)
σR_O_	σR_O_ = (1 − V_I_)/(1 − V_J_)
RC/CS_m_	RC/CS_m_ = φP_O_ × (V_J_/M_O_) × F_M_
PI_total_	PI_tatal_ = (RC/ABS) × [φP_O_/(1 − φP_O_)] × [ψ_O_/(1 − ψE_O_)] × [φR_O_/ABS(1 − φR_O_)]
V_IP_	V_IP_ = 1 − V_I_

**Table 2:** Effects of low temperature on parameters of delayed fluorescence decay curves.

Parameters	Low-Temperature Treatment		
	CK	LT2	LT4
L_1_	46.89 ± 2.03 a	37.66 ± 1.28 b	37.09 ± 1.98 b
L_2_	7.75 ± 0.7 a	7.84 ± 1.19 a	6.86 ± 0.65 a
L_3_	2.32 ± 0.41 a	1.83 ± 0.37 b	1.83 ± 0.26 b
τ_1_	0.02 ± 0 a	0.02 ± 0 a	0.02 ± 0 a
τ_2_	0.3 ± 0.05 a	0.31 ± 0.04 a	0.3 ± 0.04 a

The parameters were obtained by fitting the experimental data to the time function DF (t) = L_1_ × exp (−t/τ_1_) + L_2_ × exp (−t/τ_2_) + L_3_, with L_1_, L_2_, and L_3_ being the amplitudes (in relative units) of the kinetic components. τ_1_ and τ_2_ are lifetimes (in ms) of L_1_ and L_2_, respectively. The values are presented as means ± SD (*n* = 6). The values were presented as means ± SD (*n* = 6). The least significant difference (LSD) multiple comparison method (*p* < 0.05) was used for statistical comparisons. Different letters represent statistically significant differences (*p* < 0.05) among different treatments.

## Data Availability

Data will be made available on request.
